# Towards a toolkit for the assessment and monitoring of musculoskeletal ageing

**DOI:** 10.1093/ageing/afy078

**Published:** 2018-09-11

**Authors:** Graham J Kemp, Malcolm J Jackson, Eugene V McCloskey, John C Mathers, CIMA Toolkit Working Group, Fraser Birrell, Peter D Clegg, Daniel J Cuthbertson, Giuseppe De Vito, Jaap H van Dieën, Silvia Del Din, Richard Eastell, Patrick Garnero, Katarzyna Goljanek-Whysall, Matthias Hackl, Richard Hodgson, Sue Lord, Claudia Mazzà, Anne McArdle, Marco Narici, Mandy J Peffers, Stefano Schiaffino

**Affiliations:** 1 Department of Musculoskeletal Biology, Institute of Ageing and Chronic Disease (IACD), Faculty of Health and Life Sciences, University of Liverpool, Liverpool, UK; 2 The MRC-Arthritis Research UK Centre for Integrated Research into Musculoskeletal Ageing (CIMA), Newcastle upon Tyne, UK; 3 Mellanby Centre for Bone Research, University of Sheffield, Sheffield, UK; 4 Human Nutrition Research Centre, Institute of Cellular Medicine and Newcastle University Institute for Ageing, Newcastle University, Newcastle upon Tyne, UK

**Keywords:** musculoskeletal ageing, healthy ageing, biomarkers, skeletal muscle, bone, cartilage

## Abstract

The complexities and heterogeneity of the ageing process have slowed the development of consensus on appropriate biomarkers of healthy ageing. The MRC-Arthritis Research UK Centre for Integrated research into Musculoskeletal Ageing (CIMA) is a collaboration between researchers and clinicians at the Universities of Liverpool, Sheffield and Newcastle. One of CIMA’s objectives is to ‘Identify and share optimal techniques and approaches to monitor age-related changes in all musculoskeletal tissues, and to provide an integrated assessment of musculoskeletal function’, i.e. to develop a toolkit for assessing musculoskeletal ageing. This toolkit is envisaged as an instrument that can be used to characterise and quantify musculoskeletal function during ‘normal’ ageing, lend itself to use in large-scale, internationally important cohorts, and provide a set of biomarker outcome measures for epidemiological and intervention studies designed to enhance healthy musculoskeletal ageing. Such potential biomarkers include: biochemical measurements in biofluids or tissue samples, *in vivo* measurements of body composition, imaging of structural and physical properties, and functional tests. The CIMA Toolkit Working Group assessed candidate biomarkers of musculoskeletal ageing under these four headings, detailed their biological bases, strengths and limitations, and made practical recommendations for their use. In addition, the CIMA Toolkit Working Group identified gaps in the evidence base and suggested priorities for further research on biomarkers of musculoskeletal ageing.

Ageing is associated with the accumulation of damage to all the macromolecules within and outside cells leading to progressively more cellular and tissue defects and resulting in age-related frailty, disability and disease [1]. There is substantial inter-individual variability in the ageing process, so that biological age can differ considerably from chronological age [2]. However, the complexities and heterogeneities of the ageing process have made it difficult to define and to measure ageing, and this has slowed the development of consensus on appropriate biomarkers of ageing generically and for ageing of specific organs and tissues. Although the need has been identified [3], to date there appears to have been no attempt to develop a specific set of biomarkers of ageing of the musculoskeletal system. This Commentary provides an executive summary of a proposed toolkit for assessing musculoskeletal ageing which is published in full as a supplement to Age and Ageing [4]. This toolkit was developed by The MRC-Arthritis Research UK Centre for Integrated research into Musculoskeletal Ageing (CIMA)^1^ which is a collaboration between researchers and clinicians at the Universities of Liverpool, Sheffield and Newcastle.

## Towards a toolkit for assessing musculoskeletal ageing

One of CIMA’s objectives is to ‘identify and share optimal techniques and approaches to monitor age-related changes in all musculoskeletal tissues, and to provide an integrated assessment of musculoskeletal function’, i.e. to develop a toolkit for assessing musculoskeletal ageing. To address this objective, CIMA established a Toolkit Working Group and held a workshop with a panel of experts from UK and European institutions with well-established track records of research into musculoskeletal ageing. Workshop participants were tasked with defining a framework for the selection of biomarkers of ageing that are relevant to the multiple tissues of the musculoskeletal system, are distinct from markers of disease, change with age, and are sensitive to intervention. The ambitions for the toolkit are that it should: characterise and quantify musculoskeletal function over multiple decades, i.e. during ‘normal’ ageing, facilitate epidemiological assessment of musculoskeletal decline, provide a set of outcome measures for intervention studies (using, e.g. drugs or lifestyle) designed to enhance healthy musculoskeletal ageing and become the protocol of choice, adopted by multiple large-scale, internationally important cohorts. Candidate biomarkers were considered under four headings:

- Biochemical biomarkers
- Body composition biomarkers
- Imaging assessments
- Functional assessments

## Biochemical biomarkers of musculoskeletal ageing

Biochemical biomarkers are markers measurable *in situ* or *ex vivo* in biofluid samples and tissue biopsies that are produced in, or released from, a tissue and that are specific for a characteristic process or cell in that tissue. Two well-established serum markers of bone turnover were recommended for the toolkit i.e. N-terminal propeptide of type I procollagen (PINP) and C-terminal cross-linked telopeptide of collagen type I (CTX, also known as CTX-I). Five further markers of bone turnover viz. osteocalcin; bone alkaline phosphatase; N-terminal cross-linked telopeptide of collagen type I (NTX); carboxy-terminal cross-linked telopeptide of type I collagen generated by matrix metalloproteinases (ICTP); and tartrate-resistant acid phosphatase isoform 5b (TRACP5b) are potential future candidates. Urinary type II collagen C-telopeptide fragment (CTX-II) and serum cartilage oligomeric matrix protein (COMP) are two possible biomarkers of cartilage ageing but they have not been shown to be reliable markers of collagen ageing *per se*, independent of osteoarthritis. Serum creatinine may be a reliable biomarker of skeletal muscle mass (with appropriate dietary control). Potential next generation muscle biochemical biomarkers include: 3-methylhistidine, type VI collagen, the N-terminal peptide of procollagen type III (P3NP), agrin and growth differentiation factors.

## Body composition biomarkers

Body composition changes during development and ageing, and such changes are linked with changes in function and in the risk of age-related musculoskeletal disease. The CIMA Toolkit Working Group recommended the use of dual-energy X-ray absorptiometry (DXA) which remains the most widely recommended method for diagnosing sarcopenia (age-associated loss of muscle mass) as a stand-alone measure or as part of the screening procedure for sarcopenia and is also recommended by the European Working Group on Sarcopenia in Older People (EWGSOP) [5]. Other *in vivo* imaging techniques including ultrasound and magnetic resonance imaging have been used to measure bone and muscle volume (as a surrogate for mass) and the size of musculoskeletal structures (such as cartilage thickness), and with further development and validation may offer the ability to assess musculoskeletal tissue structure (e.g. muscle pennation).

## Functional assessments

Functional assessments test the workings of the integrated musculoskeletal system which has the advantage of direct relevance to clinical state and quality of life, for which system integration is important. The CIMA Toolkit Working Group recognised that there are several well-established functional assessment tools including the short physical performance battery (SPPB) and the locomotor domain of the NIH toolbox for assessment of neurological and behavioural function [6]. While valid and reliable tests of balance are available, and have been shown to be associated with muscle function, balance control is affected by factors other than musculoskeletal ageing *per se*. In addition, it is unclear whether balance problems precede decreases in muscle function. To date there is no fully validated system for real-life monitoring of musculoskeletal function but, in the future, low-cost body-worn movement monitors may offer an affordable and scalable solution for quantitative gait evaluation in both multicentre studies and real-world settings [7].

## Conclusions and future perspectives

Progress in developing and validating biomarkers of musculoskeletal ageing in humans has been slow and uneven and, to date, there are relatively few accepted and reliable biomarkers for ageing of this major body system. Recommendations for biomarkers of musculoskeletal ageing proposed by the CIMA Toolkit Working Group are summarised in Table 1.

**Table 1.** CIMA- recommended biomarkers of musculoskeletal ageing in humans

Biomarker	Component of musculoskeletal system assessed
PINP and CTX	Biomarkers of bone turnover
Serum creatinine	Biomarker of skeletal muscle mass
DXA	Assessment of body composition
SPPB or locomotion domain of NIH Toolbox	Assessment of physical capability

For bones and muscle, there useful biomarkers of ageing, but for the other components of the musculoskeletal system—tendons and joints—no such biomarkers are currently available. However, measures of physical capability have the advantage that, to a considerable extent, they reflect function of the integrated musculoskeletal system. This slow progress in developing biomarkers of musculoskeletal ageing parallels the situation with biomarkers of ageing *per se* [8–10], and reflects both the complexity and heterogeneity of ageing, and the difficulties in distinguishing between biomarkers of an ageing body system and biomarkers of age-related disease in that system.

## Research priorities and future development of the toolkit

This review reveals that much research effort has been devoted to disease-related biomarkers, and relatively little to biomarkers of musculoskeletal ageing itself. There are both conceptual and practical reasons for this imbalance in research focus, and the problems are amplified at older ages when multi-morbidity and polypharmacy are more common [11]. This suggests that research on biomarkers of musculoskeletal ageing is likely to be more rewarding if it is conducted earlier in the ageing trajectory. Success in the search for reliable biomarkers of musculoskeletal ageing will require innovation, not only in the application of new technologies and emerging understanding of the biology of the ageing process, but also in experimental design. There may be merit in the study of individual trajectories in musculoskeletal function during middle age, in advance of the disability and disease (including non-musculoskeletal diseases) that are likely to be major confounders. This will require repeated measures of musculoskeletal function, appropriate imaging and collection of biological samples for biomarker assessment at more frequent intervals than is usual in large ageing cohorts. In addition, measurement tools will need to be much more sensitive to detect the subtler age-related changes that are characteristic of the ageing phenotype.

We anticipate that further development of the CIMA Toolkit will include new biomarkers emerging from molecular investigation of musculoskeletal ageing. These may include DNA methylation other epigenetic-based biomarkers particularly those based on non-coding RNA species [12]. In the associated Supplement [4], we have summarised progress in identification of microRNA which are linked with risk of osteoporosis, and which may be biomarkers of joint ageing more generally. Recently, differential expression of a number of small nucleolar RNAs (snoRNA; another group of non-coding, regulatory RNA) in young versus old and normal versus OA murine joints and serum has been described, which suggests that snoRNA are also putative markers of musculoskeletal ageing [13]. In addition, a wide range of biological approaches, including proteomics and metabolomics [14], are being used to identify and validate biomarkers of ageing [15], some of which may be applicable to the musculoskeletal system.

## Footnotes

1
http://www.cimauk.org/
